# Improved estimates of 222 nm far-UVC susceptibility for aerosolized human coronavirus via a validated high-fidelity coupled radiation-CFD code

**DOI:** 10.1038/s41598-021-99204-0

**Published:** 2021-10-07

**Authors:** Andrew G. Buchan, Liang Yang, David Welch, David J. Brenner, Kirk D. Atkinson

**Affiliations:** 1 School of Engineering and Materials Science, Queen Mary University of London, E1 4NS London, UK; 2 School of Water, Energy and Environment (SWEE), Cranfield University, Bedford, MK43 0AL UK; 3 Center for Radiological Research, Columbia University Irving Medical Center, New York, NY USA; 4 Faculty of Energy Systems and Nuclear Science, Ontario Tech University, Oshawa, Ontario L1G 0C5 Canada

**Keywords:** SARS-CoV-2, Viral infection, Mathematics and computing

## Abstract

Transmission of SARS-CoV-2 by aerosols has played a significant role in the rapid spread of COVID-19 across the globe. Indoor environments with inadequate ventilation pose a serious infection risk. Whilst vaccines suppress transmission, they are not 100% effective and the risk from variants and new viruses always remains. Consequently, many efforts have focused on ways to disinfect air. One such method involves use of minimally hazardous 222 nm far-UVC light. Whilst a small number of controlled experimental studies have been conducted, determining the efficacy of this approach is difficult because chamber or room geometry, and the air flow within them, influences both far-UVC illumination and aerosol dwell times. Fortunately, computational multiphysics modelling allows the inadequacy of dose-averaged assessment of viral inactivation to be overcome in these complex situations. This article presents the first validation of the WYVERN radiation-CFD code for far-UVC air-disinfection against survival fraction measurements, and the first measurement-informed modelling approach to estimating far-UVC susceptibility of viruses in air. As well as demonstrating the reliability of the code, at circa 70% higher, our findings indicate that aerosolized human coronaviruses are significantly more susceptible to far-UVC than previously thought.

## Introduction

One year into the COVID-19 pandemic, active measures such as mask wearing^1^ and social distancing continue to play a dominant role in preventing the spread of SARS-CoV-2. Whilst vaccination efforts have commenced across the globe, supply chain issues^2^, adjustment of vaccination regimens^3–5^, vaccine hesitancy^6–8^, and uncertainty over whether current vaccines give sufficient protection against variant strains^9–13^ all challenge the aim of reaching herd immunity. Consequently, when opening up their high-traffic public spaces like airport terminals, restaurants and gyms with an abundance of caution, local governments, companies and organisations have upgraded heating, ventilation and air conditioning (HVAC) systems, often with HEPA filters or ultraviolet germicidal irradiation (UVGI)^14–19^. In schools, offices and long-term care facilities where infrastructure is often outdated or suffering from under-investment, use of in-room air purifier devices has been commonplace^20–22^. Whilst all these efforts should be commended, recent work has shown that the efficacy of these measures may not be as good as expected because the air currents stirred up by these devices can cause virus-contaminated air to be pulled past the faces of people in these spaces, may lead to stalled air flow in some parts of the room, and may even promote resuspension of virus-containing droplets^23–26^. In-room, 222 nm filtered far-UVC illumination has been suggested as a potential alternative approach, wherein aerosolized viruses are inactivated whilst transported in air flows within a room^27^. The efficacy of this approach was predicted for a hospital room via use of the WYVERN high-fidelity coupled radiation-CFD code^28^. Several major companies now supply far-UVC lamps. Benchtop studies at Columbia University have successfully demonstrated that, with a commercially available lamp, far-UVC light does inactivate aerosolized HCoV-229E and HCoV-OC43 human coronaviruses^29^. However, to demonstrate similar efficacy in more realistic settings and general rooms is more challenging. Whilst they may share similar features, each room is unique. Placement of lamps, HVAC, doors, windows and furniture will all influence air flows and illumination and hence a single experimental test is not adequate to predict efficacy in a reliable manner for all but the most simple cases. Indeed, even the movement of people in a room will disturb the air flow within it^30^. This obviously has implications for risk assessment in the workplace or spaces that can be accessed by members of the public; without adequate quantification of efficacy, use of far-UVC lamps cannot form part of an infection risk mitigation strategy. As the biosafety hazard makes measurements of aerosolized viruses impractical outside of a specialized aerobiology facility, it is not feasible to build laboratory representations of every possible room, nor to test efficacy in-situ at this point in time. Computational modelling provides perhaps the only viable solution. Able to capture the geometry of any room, along with any perturbing factors that will influence illumination and air flow, it does not suffer from the same constraints as practical measurements and allows more detailed analysis to be conducted, e.g. to ensure that far-UVC lamps are positioned optimally. Obviously, to have confidence in computational predictions, any code should be able to accurately replicate the results seen in the aforementioned benchtop studies, along with any large-scale experimental investigations that may follow.

**Figure 1:**
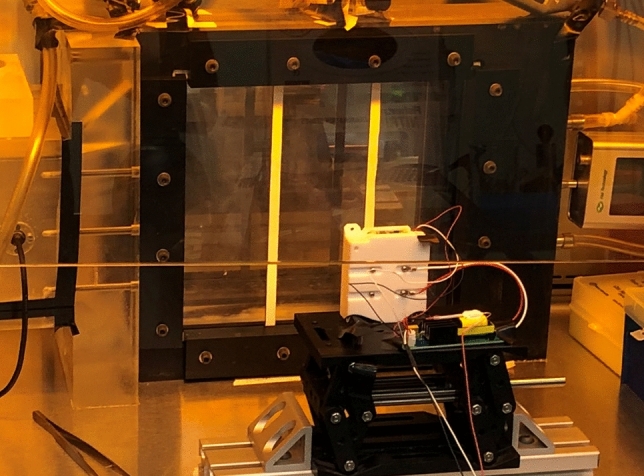
The experimental configuration at Columbia University had a flow-through aerosol irradiation chamber with a 222 nm filtered far-UVC lamp placed 22 cm away.

One element of this study addresses the need for methods validation by applying the WYVERN high-fidelity coupled radiation-CFD code to reproduce the experimental measurements of 222 nm UVC inactivation of aerosolized coronavirus made at Columbia University^29^. The experimental apparatus used at Columbia University is shown in Fig. 1, the full dimensions and configurations of which can be found in the literature^27,29^. The basic setup consisted of a far-UVC irradiation chamber of size $26 \times 6.3 \times 25.5 \ {\text {cm}}$ through which human coronavirus-bearing aerosol was pumped at a rate of 12.5 L/min and thereafter collected for analysis through two small outlet pipes. The source of 222 nm far-UVC light was a single lamp placed 22 cm from the chamber, the light entered the chamber through a polymer film window that reduced far-UVC intensity by 35%. The back wall of the chamber was fitted with an aluminium plate that reflected around 15% of the far-UVC light. Two 1 cm wide vertical support rods provided obstructions that prevented some light from reaching the chamber volume. For the given configuration it was estimated that the aerosolized virus would take approximately 20 seconds to transit the chamber and hence, as the average fluence rate was estimated to be 0.1 $\hbox {mJ}.\hbox {cm}^{-2}.\hbox { s}^{-1}$, an average dose of 2 $\hbox {mJ}.\hbox {cm}^{-2}$ was absorbed by the coronavirus virions. Control over the fluence rate in the chamber was facilitated through placement of 222 nm far-UVC absorbing polymer films, held in place by the support rods, in front of the chamber window. These filters transmitted 50% and 25% of the incident far-UVC light, respectively, allowing further measurements to be made at estimated doses of 1 and 0.5 $\hbox {mJ}.\hbox {cm}^{-2}$. Inactivation of the aerosolized coronavirus was evaluated via a virus infectivity assay. Assuming that both the far-UVC radiation field and the aerosol fluid velocity were uniform throughout the chamber; from the measured survival of aerosolized HCoV-229E and HCoV-OC43 human coronaviruses, 222 nm far-UVC susceptibilities, Z, were deduced. For HCoV-229E the estimated 222 nm far-UVC susceptibility was calculated to be 4.1 $\hbox {cm}^{2}\hbox {mJ}^{-1}$with a 95% confidence interval from 2.5 to 4.8 $\hbox {cm}^{2}\hbox {mJ}^{-1}$. For HCoV-OC43 the estimated Z was 5.9 $\hbox {cm}^{2}\hbox {mJ}^{-1}$ with a 95% confidence interval from 3.8 to 7.1 $\hbox {cm}^{2}\hbox {mJ}^{-1}$.

The estimates of viral susceptibility to UVC derived from experiment were based on the idealised assumption of uniform airflow and radiation intensity. In reality this is incorrect because the radiation intensity varied due to the light source being non-uniform (see methods section) and because there were shadows cast by the obstructing support structures. The flow velocities are also non-uniform due to the frictional forces around the chamber walls, and due to the flow’s re-direction towards the narrow outlet tubes. The validity of the assumptions used to calculate Z should therefore be questioned since variation in the far-UVC or fluid fields would cause noticeable variations in aerosolized virus exposure times, and hence have direct implications with respect to the calculation of far-UVC susceptibility. This article therefore addresses this need for a complete understanding of the physical behaviour within the irradiation chamber before far-UVC susceptibilities are attributed. It does this by presenting the first highly-detailed model of the spatially varying flow fields and far-UVC fluence rates within the chamber, and provides the first fully-validated computational model of the far-UVC inactivation of aerosolized human coronavirus in a real system. Importantly this leads to the first implementation of a combined modelling-experimental approach to the improved estimation of far-UVC susceptibility. Both were achieved by using the WYVERN coupled radiation-CFD code^28^, a unique high-fidelity modelling capability employing Boltzmann radiation transport and Large Eddy Simulation (LES)-Navier-Stokes descriptions of respective physics domains.

## Results

**Figure 2:**
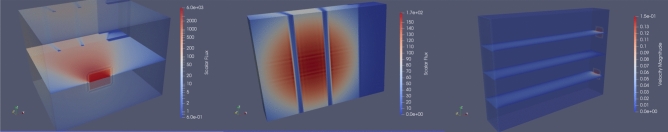
Left: Far-UVC intensity (the fluence rate, $\upmu \hbox {J}.\hbox {cm}^{-2}.\hbox {s}^{-1}$) in the region spanned from the lamp surface to the back wall of the chamber. The high intensity red rectangular region in the foreground denotes the position of the lamp. Middle: Far-UVC intensity within the chamber. The vertical bars indicate the shadows cast by the support rods. Right: Fluid velocity magnitudes for developed aerosol flow, from left to right, within the chamber.

The predicted far-UVC intensity fields and aerosol flow velocities within the irradiation chamber are presented in Fig. 2. The radiation model was tuned to the characteristics of the Care222 filtered far-UVC lamp (Ushio, Inc.)^29,31^, the type of lamp used in the experiments, to correctly describe the anisotropic source distribution created by the filter and lamp housing. The details of the source model are presented below. The 222 nm far-UVC intensity inside the chamber exhibits considerable variation due to the angular distribution of the light source, caused by the filter and lamp housing, and the shadows cast by the aforementioned vertical support rods. The highest far-UVC intensities were found in the mid-section of the chamber where they reached a maximum of 0.15 $\hbox {mJ}.\hbox {cm}^{-2}.\hbox { s}^{-1}$, but dropped to around 0.035 $\hbox {mJ}.\hbox {cm}^{-2}.\hbox { s}^{-1}$ in the corners. The mean far-UVC intensity within the chamber was found to be 0.081 $\hbox {mJ}.\hbox {cm}^{-2}.\hbox { s}^{-1}$, 19% smaller than the original estimate^29^. The fully-developed aerosol flow field also showed spatial variations, higher fluid velocities forming in the centre of the chamber due to the drag forces on the chamber walls and the positioning of the outlet tubes. Human coronavirus was simulated with a particle model that represented the virus-bearing aerosols moving with the flow field. Once the simulated flows had fully developed within the running code, aerosols were released at points on a $40\times 40$ grid evenly distributed across a $6.1 \times 25.1$ cm area on the face of the inlet and their migration tracked through the irradiation chamber. The viral inactivation within each particle was then calculated by integrating the far-UVC fluence received at each respective position and from this, once all particles had exited the chamber, an overall survival fraction was calculated. A far-UVC susceptibility of 4.1 $\hbox {cm}^{2}\hbox {mJ}^{-1}$ was assumed^28,29^.

**Figure 3:**
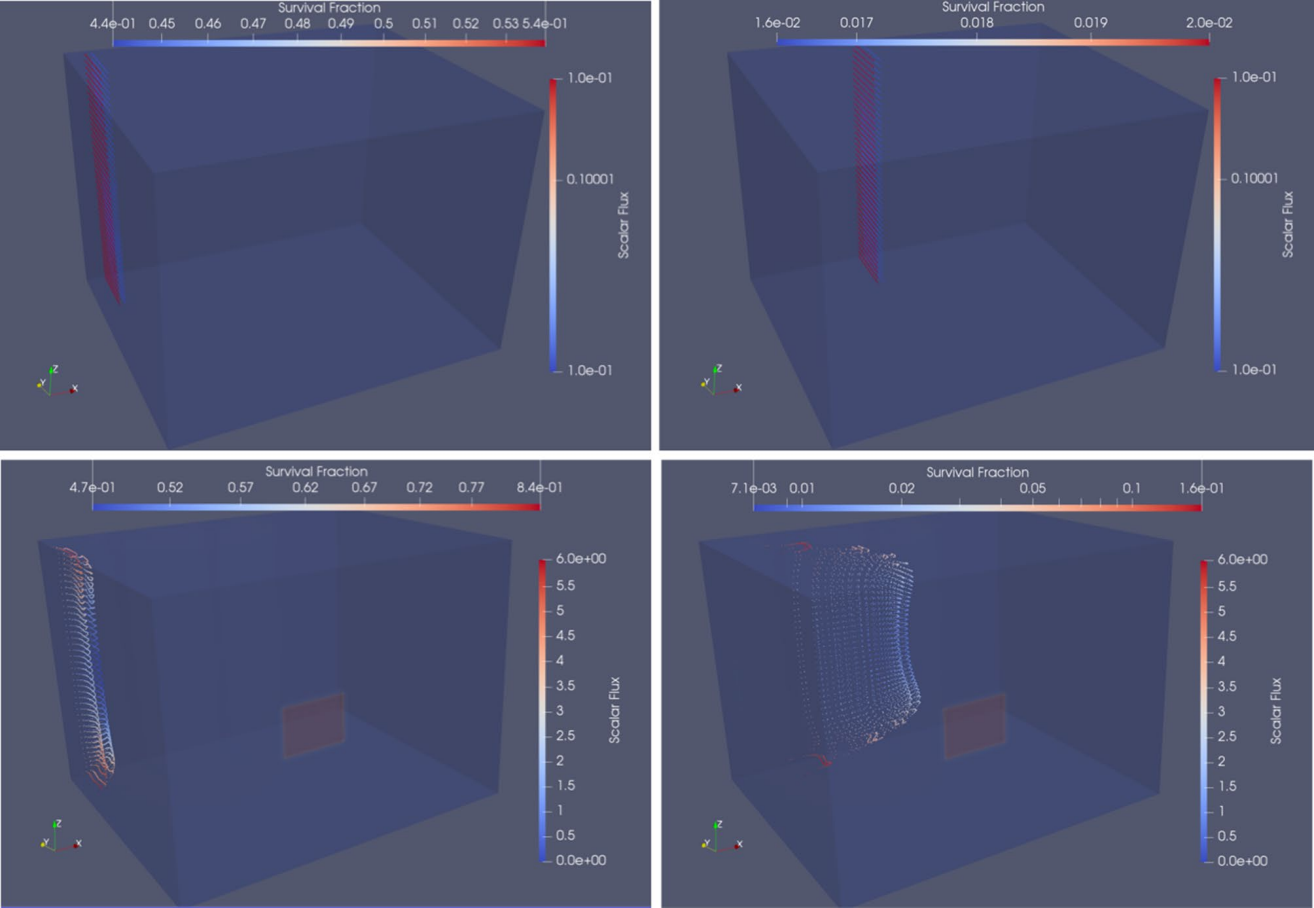
Aerosol distribution within the irradiation chamber and human coronavirus survival fraction at 2 (left) and 10 (right) seconds. Top: predictions when uniform flow and far-UVC intensity are assumed, Bottom: predictions using CFD-flow and model-predicted far-UVC radiation fields. Particle colour denotes survival. Assumed far-UVC intensities are superimposed.

**Figure 4:**
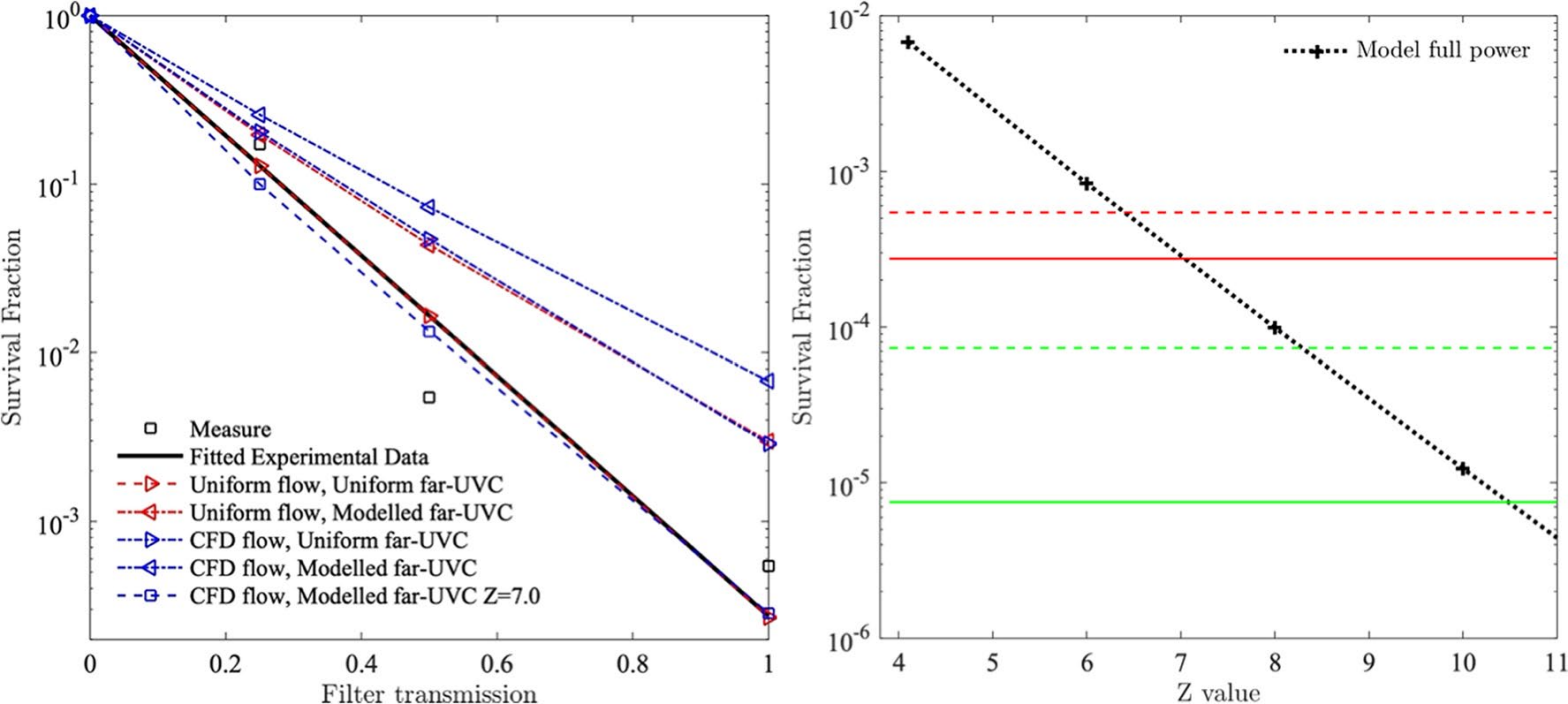
Left: Survival fractions as a function of filter transmission for HCoV-229E. A transmission of 1 assumes no additional filtration was installed in the lamp-chamber system. All of these modelling results utilized the previously calculated Z value of 4.1 $\hbox {cm}^{2}\hbox {mJ}^{-1}$ except for the condition with $Z= {7.0}$ $\hbox {cm}^{2}\hbox {mJ}^{-1}$ which is the predicted correct value of Z using the model. Transmissions of 0.5 and 0.25, respectively, represent 50% and 75% attenuation of the far-UVC lamp intensity due to installation of additional 222 nm-absorbing polymer films. Right: Survival fractions estimated for varying Z values predicted by the model. Comparison against measured data for HCoV-229E (red) and HCoV-OC43 (green). Dashed line: measured data survival fractions. Solid line: fitted data survival estimates.

The model was run, and the results analysed, for four sets of flow conditions and far-UVC intensity fields. The first took the original conditions assumed by the experimenters at Columbia University^29^, namely a uniform far-UVC intensity of 0.1 $\hbox {mJ}.\hbox {cm}^{-2}.\hbox { s}^{-1}$ and a uniform flow velocity of $1.3\,\hbox {cm.}\hbox { s}^{-1}$. The second and third combinations utilised either the model-predicted far-UVC radiation field, or the model-predicted flow field, respectively, whilst keeping the other field uniform. The fourth combination used both model-predicted fields. Figure 3 shows the distribution of virus-bearing aerosols within the chamber and the predicted inactivation of human coronavirus for the different flow and radiation conditions at two time points. It was observed that, along with the separation of particles due to variations in the flow velocity, there was decreased coronavirus inactivation in regions close to the upper and lower sections of the chamber due to reduced far-UVC intensity. The survival fractions predicted by the model are presented in Fig. 4 alongside the original measured data points and the curve fitted to these data^29^. The simulation using uniform far-UVC intensity and fluid velocities match almost exactly the fitted data, as would be expected since all assumed conditions are identical. When CFD-modelled flow was considered, but far-UVC intensity kept uniform, there was a considerable increase in predicted surviving fraction. This can be attributed to there being regions within the chamber with fast fluid velocities, forcing virus-bearing aerosols through more quickly, reducing their dwell times in the far-UVC field, and therefore reducing overall coronavirus inactivation. A similar increase in surviving fraction was seen for the case of uniform aerosol flow through the model-predicted radiation field. Here, whilst the flow was slower and hence the aerosol dwell times longer, the far-UVC intensity was lower (by around 19% on average) and therefore overall viral inactivation was reduced. Finally, for the case of CFD-modelled flow and a model-predicted radiation field; shorter dwell times in some regions of the chamber coupled with lower far-UVC intensity causes the surviving fraction to be greater still. Clearly when the high-fidelity coupled radiation-fluid model was used to represent the physical behaviour within the irradiation chamber, assuming the far-UVC susceptibilities quoted by Buonanno et al.^29^ does not lead to predictions of aerosolized human coronavirus survival comparable to those measured in experiments.

As the code precisely reproduced the published survival curves of Buonanno et al.^29^ when considering their estimated Z values and uniform field assumptions, it was apparent that better informed estimates of far-UVC susceptibility could be made by determining Z values at which equivalent survival was found for detailed fluid velocity and far-UVC intensity field conditions. Figure 4 (right) shows the modelled survival fraction as a function of Z when the detailed CFD-modelled flow and model-predicted radiation field were considered. Inactivation was simulated assuming a filter transmission of unity, i.e. the was no additional filtration, for a range of Z values. Superimposed on the graph are horizontal lines representing the as-measured and fitted survival fractions deduced by Buonanno et al.^29^ for HCoV-229E and HCoV-OC43 exposed to the same far-UVC intensity field. Points of intersection show the corrected value of Z as guided by the model. By taking the intersects with the fitted survival fractions, Z values of 7.0 $\hbox {cm}^{2}\hbox {mJ}^{-1}$ and 10.5 $\hbox {cm}^{2}\hbox {mJ}^{-1}$ were estimated for HCoV-229E and HCoV-OC43, respectively. Taking the improved estimate for the 222 nm susceptibility of HCoV-229E, the simulations with the CFD-modelled flow and model-predicted radiation fields were repeated for filter transmissions of 0.5 and 0.25. The results, plotted on Fig. 4 (left), show very close agreement to those found from the fitted data. A similar agreement was found for HCoV-OC43 when the improved Z value was used.

## Discussion

Due to the unique nature of each possible installation, generalised claims about the efficacy of germicidal irradiation of aerosolised coronavirus using in-room 222 nm far-UVC lamps cannot be made. As it is not tenable to make real-world measurements or to build infinite numbers of mock ups for testing purposes, only computational modelling and simulation approaches provide a universal tool for assessing the efficacy of in-room far-UVC for infection control. As with all computational modelling, code validation against available measurements is essential for trustworthy predictions of real-world applications. In this article the WYVERN high-fidelity coupled radiation-CFD code has been validated against benchtop measurements of aerosolized human coronavirus inactivation in a flow-through far-UVC irradiation chamber. As part of the exercise, the radiation transport model was separately validated against, and shown to be in close agreement with, measured data^32^ for the type of lamp used in the experiment. This provided necessary assurances of accurate predictions of the far-UVC fluence within the chamber. Whilst other codes could be used to model the experiments, this represents the first time that a computer code has been validated for this specific purpose. Moreover, it highlighted the complications encountered when trying to determine susceptibilities to 222 nm far-UVC, or indeed any UV, light from inactivation measurements of aerosolized viruses.

Unlike surface inactivation experiments wherein uniform illumination can be facilitated, in experiments that involve moving air flows with varying velocities and far-UVC intensities that change across the domain, assuming uniform exposure of virus-bearing aerosol particles is not a satisfactory approach to assessing survival unless sufficient mixing can be encouraged. Clearly, any heterogeneity of exposure caused by varying dwell times or far-UVC fluence rates, will mean that accurate determination of fluence is hindered. The uncertainty in attributing total dose received is reflected in Fig. 4 (left) wherein values were plotted against filter transmission, a constant condition under all in-chamber situations, rather than dose. Obtaining accurate descriptions of both the flow field and far-UVC intensity through measurement alone is extremely challenging. This article has shown that high-fidelity computational models can be used to provide descriptions of physical conditions not adequately sampled via measurements and that, when considered, the effect of these conditions can be significant. For this experiment, fully accounting for the variations in aerosol flow velocity and far-UVC intensity meant that agreement with measured survival fractions necessitated the use of improved far-UVC susceptibility values, i.e. 7.0 $\hbox {cm}^{2}\hbox {mJ}^{-1}$ and 10.5 $\hbox {cm}^{2}\hbox {mJ}^{-1}$ for HCoV-229E and HCoV-OC43, respectively. Clearly, our findings indicate that aerosolized human coronaviruses, and by extension SARS-CoV-2, are significantly more susceptible to 222 nm far-UVC than previously thought.

## Methods

The survival *S* of a viral population subjected to some intensity (fluence rate) of UVC radiation over a time period of *t* seconds is governed by the equation,

1 $$\begin{aligned} S =e^{-Zd} =e^{-ZE_pt}, \end{aligned}$$

as described in^33^. The UVC intensity of dimensions $\hbox {mJ}.\hbox {cm}^{-2}.\hbox { s}^{-1}$is denoted by $E_p$, and the dose (fluence) received (with units $\hbox {mJ}.\hbox {cm}^{-2}$) is denoted by $d = E_p t$. The key parameter governing the rate of viral inactivation is the susceptibility, Z, of units $\hbox {cm}^{2}\hbox {mJ}^{-1}$. Susceptibility is dependent on both the virus type, its host media (e.g. air, water), and the UVC wavelength. Relating to SARS-CoV-2 estimates of *Z* have been provided in^29^ which states a value 4.1$\hbox {cm}^{2}\hbox {mJ}^{-1}$ for aerosol conditions.

### Far-UVC Radiation Transport Model

The intensity of the far-UVC field is described through the mono-energetic, fixed-source Boltzmann transport equation,

2 $$\begin{aligned} \varvec{\Omega }\cdot \varvec{\nabla } E(\varvec{r},\varvec{\Omega })+\Sigma _{t}(\varvec{r}) E(\varvec{r},\varvec{\Omega }) - \int _{\varvec{\Omega }'} \Sigma _{s}(\varvec{r},\varvec{\Omega }' \rightarrow \varvec{\Omega }) E(\varvec{r},\varvec{\Omega }') d \varvec{\Omega }' = S(\varvec{r}, \varvec{\Omega }). \end{aligned}$$

The radiation intensity distribution $E(\varvec{r},\varvec{\Omega })$ exists within a five-dimensional phase-space consisting of three space dimensions, $\varvec{r}$, and two angles $\varvec{\Omega }$, and has units of $\hbox {mJ}.\hbox {cm}^{-2}.\hbox { s}^{-1}$. The equation describes the transport of far-UVC photon energy and accounts for photon interactions with surrounding media through absorption and scattering characterised by the cross-sections $\Sigma _t(\varvec{r})$ and $\Sigma _s(\varvec{r})$, respectively. Any volume source of far-UVC is described through the term $S(\varvec{r}, \varvec{\Omega })$, which, in this article is set to zero. Incoming boundary conditions applied to the exterior surface of the problem are denoted by $\Gamma$, and include surface sources,

3 $$\begin{aligned} E(\varvec{r},\varvec{\Omega }) = S(\varvec{\Omega }) \ , \varvec{n} \cdot \varvec{\Omega } < 0 \ , \varvec{r} \in \Gamma \end{aligned}$$

and diffuse, or white, reflecting surfaces,

4 $$\begin{aligned} E(\varvec{r},\varvec{\Omega }) = \alpha _{r} \frac{ \int _{ \varvec{\Omega }^{'} \cdot \varvec{n}>0 } ( \varvec{\Omega }^{'} \cdot \varvec{n} ) E(\varvec{r},\varvec{\Omega }^{'}) d \varvec{\Omega }^{'} }{ \int _{ \varvec{\Omega }^{'} \cdot \varvec{n} >0 } ( \varvec{\Omega }^{'} \cdot \varvec{n} ) d \varvec{\Omega }^{'}} \ \ \ \ \ \ \varvec{n} \cdot \varvec{\Omega } < 0 \ , \varvec{r} \in \Gamma . \end{aligned}$$

The term $\varvec{n}$ denotes the outward pointing normal from the surface and $\alpha _{r}$ the reflecting albedo. For a surface position occupied by the lamp the function $S(\varvec{\Omega })$ takes the form of the incoming angular distribution as projected by the lamp. If a void, or vacuum, condition is applied, equation 3 is used with $S(\varvec{\Omega })=0$.

The solution to Eq. (2) was obtained via a model using discontinuous finite elements and discrete ordinates for resolving the spatial and angular dimensions respectively. The solutions presented included the entire domain encompassing the lamp, irradiation chamber and exit region that spanned a cuboid of dimension $34.5 \times 28.5 \times 25.5$ cm. A uniform mesh of 0.5 cm$^3$ hexahedral elements with linear basis functions was employed together with a high order $S_{100}$ angular discretisation of the angular domain. In 3D this used 10200 directions which provided sufficient resolution to cover the experimental domain with far-UVC without any complications due to ray-effects. This space-angle discretisation resulted in a total of 16.3 billion degrees of freedom for the whole radiation calculation.

The scalar quantity of the spatially dependent far-UVC intensity, $E_p(\varvec{r})$, that irradiates aerosolized (or airborne) virus was obtained by integrating over the angular dimensions of the intensity variable,

5 $$\begin{aligned} E_p(\varvec{r}) = \int _{\varvec{\Omega }} E(\varvec{r},\varvec{\Omega }) d \varvec{\Omega }. \end{aligned}$$

As determined in^28^ the interaction of 222 nm far-UVC in air is extremely low and hence material cross-sections of $\Sigma _t = 1 \times 10^{-5}$ were used.

### Fluid flow model for room or chamber ventilation

Computational fluid dynamics is a numerical approach for simulating the movement of air based on the conservation laws of mass, momentum, and energy. Ignoring the temperature influences, the airflow motion is governed by the following form of the unsteady, incompressible Navier-Stokes equations:

6 $$\begin{aligned} \varvec{\nabla } \cdot \varvec{u}&= 0 , \nonumber \\ \varvec{u}_t+\varvec{u}\cdot \varvec{\nabla }\varvec{u} + \varvec{\nabla } p - \nu {\nabla }^2 \varvec{u}&= 0 . \end{aligned}$$

The velocity of air is denoted by the three-component vector $\varvec{u}=(u,v,w)$ which holds the respective air velocities in the x, y and z dimensions, and *p* denoting the pressure. The kinematic viscosity of air is denoted by $\nu$ and has the value $1.5 \times 10^{-5}\hbox {m}^{2}.\hbox {s}$. The Reynolds number ($Re=\frac{U L}{\nu }$ ) for this problem was under a laminar flow regime.

In the simulations presented, a finite element discretisation of the governing Eq. (6) was used^34^. A regular mesh of 0.5 cm$^3$ hexahedral elements was employed upon which both the velocities and pressures were resolved using continuous linear basis functions. The transient process was resolved using the explicit Adams–Bashforth stepping scheme. A Large Eddy Simulation was embedded in the fluid solver for resolving the flows’ turbulent features, albeit this was not required for the experiment herein due to it having a low Reynolds number flow. The full details of the finite element discretisation of the Eqs. (6-8) and the LES model are discussed in^34,35^.

### Lagrangian Nuclei model

The trajectory of aerosol particles was described by the balance equation. The velocity $\varvec{v}_i$ of each particle, *i*, being determined through the equation,

7 $$\begin{aligned} \frac{d \varvec{v}_i}{dt} = F_d (\varvec{u}_i-\varvec{v}_i) + g (1-\frac{\rho }{\rho _a}) + f_{i,Brownian} , \end{aligned}$$

which relates its change in velocity due the surrounding fluid velocity $\varvec{u}_i$, and gravity *g*. The right-hand term in Eq. (7) represents the Stokes drag force, gravity and the Brownian force. The model is capable of resolving the turbulent dispersion of particles, and the random effects of turbulence on particle dispersion using the discrete random walk method - the last term in Eq. (7). However, due to the short timescales spent in the irradiation chamber and the small droplet size, the effects of gravity were neglected. The particles were initiated at the inlet on the left-hand side of the chamber. These were uniformly distributed across the inlet face as a set of particles in the form of a 40 $\times$ 40 grid. In total 3 sets of particles were released every 0.2 seconds once the velocity flow field had fully developed.

### UVC inactivation model

Associated with each particle is a scalar field representing the surviving fraction of any given virus contained within it. Initialised with a value of unity, the surviving fraction, $\phi _i$, of particle *i* is governed by the equation,

8 $$\begin{aligned} \frac{d \phi _i}{dt} = ZE_p\phi _i - \alpha \phi _i , \end{aligned}$$

Inactivation of virus is governed by its irradiation in the far-UVC field which is resolved by the first term in the right-hand side of Eq. (8), involving multiplication by the far-UVC intensity (fluence rate), $E_p$, at the particles’ position, and the susceptibility *Z* of the virus. The natural inactivation (death) rate, or half life, of the virus has been included in the second term of Eq. (8), wherein $\alpha$ denotes the death rate. However, due to the short timescale for the experiment this was insignificant and hence not considered.

### Validation of the far-UVC model for predicting illumination from a Care222 filtered far-UVC lamp

For completeness, this section provides a validation of the radiation transport model within the WYVERN code to confirm that it predicts the correct intensities when modelling the Care222 filtered far-UVC lamp used in the experiment^29^ (Item #9101711, Cypress, CA). A simulation was performed to predict the illumination from the lamp in an air-filled box of size 1.1 $\hbox {m}^3$. The box was illuminated by a single 12 W lamp of approximate surface area $4.5 \times 6.0$ cm, positioned in the top corner of the domain. An important characteristic of the lamp is that its bandwidth-limiting filter produces a highly anisotropic, forward-peaked angular-distributed light source. It is essential that this source is fully and accurately resolved for accurate prediction of illumination across the entire domain.

**Figure 5:**
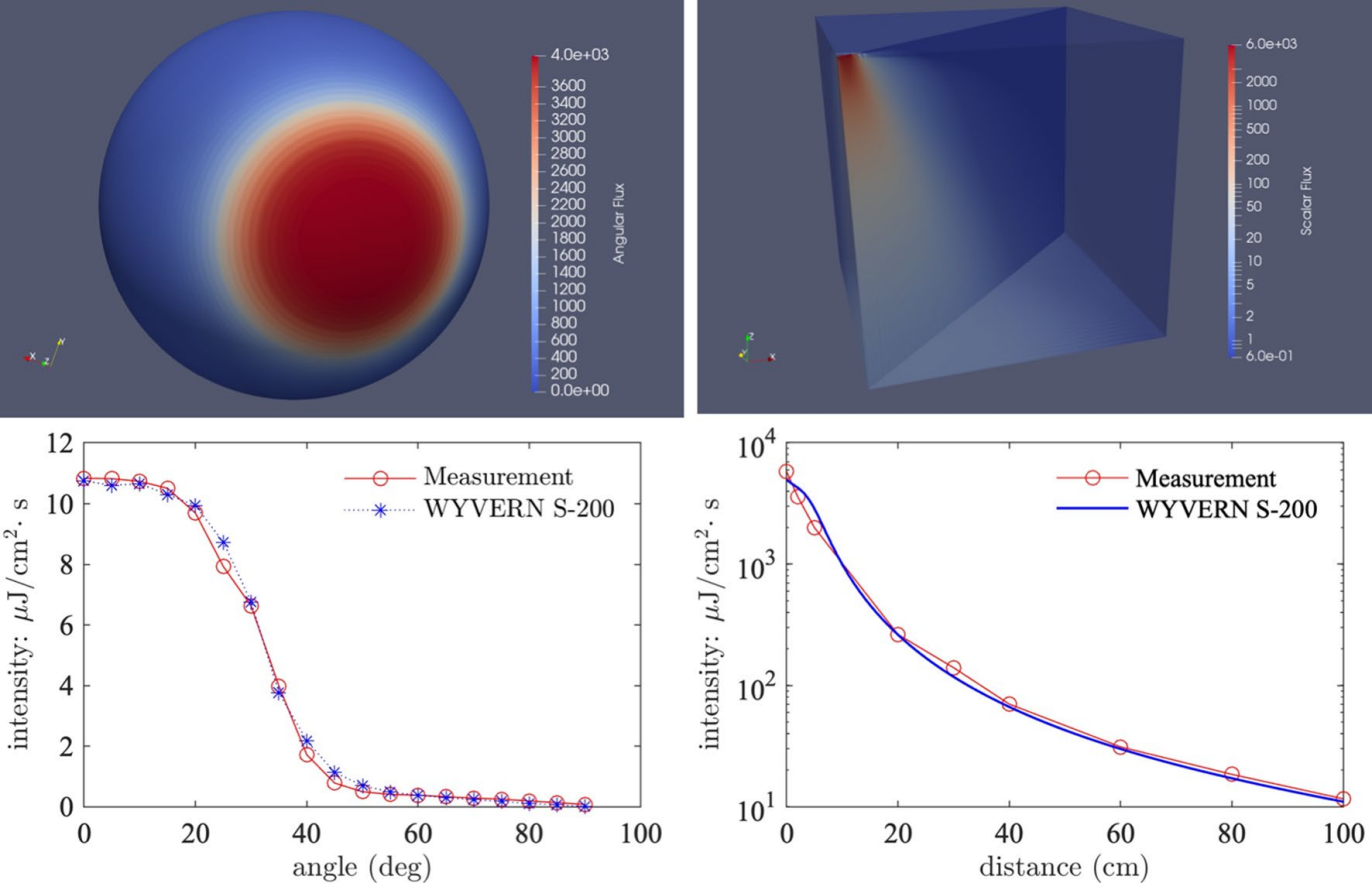
Top left: Angular flux surface source intensity, the value at the spherical surface represents the flux intensity in the direction defined by the angle from the origin of the sphere to its surface. Top Right: Far-UVC scalar flux intensity (fluence rate) through space in units of $\upmu \hbox {J}.\hbox {cm}^{-2}.\hbox {s}^{-1}$. Bottom Left: Far-UVC intensity (fluence rate) as a function of angle at a distance of 1 mfrom the light source. Bottom Right: Far-UVC intensity (fluence rate) as a function of distance from the light source.

The WYVERN code has the ability to resolve arbitrary angular-distributed surface sources and hence was tuned to available data measurements, the angular flux profile of the surface source, as generated by the model, is presented in Fig. 5 (top left). The model used a uniform spatial mesh of $1 \times 0.5 \times 1$ cm$^3$ hexahedral finite elements and an $S_{200}$ discretisation, totalling 860 billion degrees of freedom, and the predicted light intensity through the domain is presented in Fig. 5 (top right). The validation rests on agreement between the model, the experimental measurements^29^, and the graph^32^ presented in Fig. 5 (bottom left) showing the predicted intensity 1 mdistance from the lamp as a function of angle with respect to the face of the source. The graph in Fig. 5 (bottom right) shows the model-predicted far-UVC intensity as a function of distance up to 1 m from the lamp. In both cases there is close agreement between the simulated and measured data confirming the model is capable of accurately reconstructing the anisotroptic flux (fluence rate) characteristics caused by the lamp filter.
